# Distinct rhizosphere soil responses to nitrogen in relation to microbial biomass and community composition at initial flowering stages of alfalfa cultivars

**DOI:** 10.3389/fpls.2022.938865

**Published:** 2022-08-24

**Authors:** Yunru An, Haoyang Sun, Wei Zhang, Yunfu Sun, Shuxia Li, Zhouchang Yu, Rongchen Yang, Tianming Hu, Peizhi Yang

**Affiliations:** ^1^College of Grassland Agriculture, Northwest A&F University, Xianyang, China; ^2^College of Agricultural, Ningxia University, Yinchuan, China

**Keywords:** alfalfa cultivars, soil properties, rhizosphere soil microbial community, microbial diversity, soil nitrogen

## Abstract

In the long-term growth process, alfalfa rhizosphere forms specific microbiome to provide nutrition for its growth and development. However, the effects of different perennial alfalfa cultivars on changes in the rhizosphere soil characteristics and microbiome are not well understood. In this study, 12 perennial alfalfa cultivars were grown continuously for eight years. Rhizosphere samples were tested using Illumina sequencing of the 16S rRNA gene coupled with co-occurrence network analysis to explore the relationship between alfalfa (biomass and crude protein content), soil properties, and the microbial composition and diversity. Redundancy analysis showed SOC and pH had the greatest impact on the composition of the rhizosphere microbial community. Moreover, microbial diversity also contributes to microbial composition. Soil properties (AP, EC, SOC and pH) exhibited a significant positive correlation with soil bacterial communities, which was attributed to the differences between plant cultivars. Partial least squares path modeling (PLS-PM) revealed that microbial biomass and community composition rather than diversity, are the dominant determinants in the rhizosphere soil nitrogen content of perennial alfalfa. Our findings demonstrate that the soil microbial biomass and composition of rhizosphere bacterial communities are strongly affected by cultivar, driving the changes in soil nitrogen content, and variances in the selective capacities of plants.

## Introduction

Alfalfa (*Medicago sativa* L.) is a perennial forage legume with high yield and good nutritional quality that is widely considered important and cultivated (Annicchiarico, 2015). Owing to the high value in nutrients beneficial to livestock performance, alfalfa is known as “the queen of forage crops”(Hrbackova et al., 2020). The quality of alfalfa is mainly based on its protein content and fiber which affect animal feed digestibility and nutrient intake (Yang et al., 2021). Alfalfa being legume plants can fix nitrogen naturally through symbiotic nitrogen fixation (Fonouni-Farde et al., 2016). This characteristic can affect the content of crude protein, biomass of alfalfa plants, and soil N content. The crude protein content of alfalfa is relatively high (over 20% in its stems and leaves; Elfaki and Abdelatti, 2016), with significant differences generally found among different cultivars (Wang et al., 2022).

The rhizosphere is a critical hotspot for biogeochemical transformation and harbors one of the most complex, diverse, and active plant-associated microbial communities (Zhalnina et al., 2018), which is strongly influenced by both roots and the surrounding soil (Tkacz et al., 2020a). Rhizosphere effect describes the enrichment and activity of microbial cells near growing roots (Cúcio et al., 2016; Sasse et al., 2018). Previous studies have reported that some microbes are parasitic in plants and can interact with each other (Shelake et al., 2019). Rhizosphere microorganisms can also be recruited by the plant host to perform essential roles that contribute to soil nutrient cycling, healthy root growth, and plant community development in the soil ecosystem (Song et al., 2019). Therefore, plant growth and development are strongly affected by the responses of the rhizosphere microbial community, as it contributes directly in nutrient acquisition, defense, and abiotic stress responses (de Faria et al., 2021). Bulk soil is the main source of microbes colonizing the root rhizosphere, and plant genotype partially drives the selection of rhizosphere microorganisms (Mendes et al., 2018b). As a rich source of nutrients for soil microorganisms, plants affect the input and metabolism of microbial nutrition owing to differences in the distribution of core rhizosphere microorganisms of each plant variety (Zhang et al., 2021). Hence, understanding the influence of rhizosphere soil characteristics through differences in alfalfa cultivars is important for improving animal husbandry development and the soil N cycle (Samaddar et al., 2021).

The diversity and abundance of bacteria in plant rhizosphere soils are affected by several factors. Plants release root exudates, which significantly influence microbial responses, root architecture, and nutrient uptake (Wen et al., 2021). Cultivars can differ greatly in root exudate structure and release patterns, and therefore can strongly affect microbial activity and community structure. Previous reports have shown that changes in soil microbial diversity and structure are depend on plant species, soil physiochemical characteristics, and land use types (Mitchell et al., 2010; Brereton et al., 2020; Rossmann et al., 2020; Tkacz et al., 2020b; Xu et al., 2020). Soil bacterial communities are shaped by several soil factors (Llado et al., 2018), especially pH (Mod et al., 2021), along with other edaphic factors such as soil electrical conductivity (EC), soil organic carbon (SOC; Yashiro et al., 2016), total and available phosphorus (Dai et al., 2020). The diversity, composition, and biomass of soil microbial communities play key roles in nutrient acquisition, cycling, and movement patterns. Soil microbial biomass is a reserve of soil nutrients and has an excellent ability to decompose organic matter and convert complex N-rich organic matter into ammonia (Tao et al., 2019). Therefore, any changes in microbial responses can strongly affect crop growth.

A previous study reported the composition of naturally occurring rhizosphere bacterial populations and their metabolic activities differed between transgenic and unmodified alfalfa plants grown in the field (Tesfaye et al., 2003). While long-term planting of switchgrass did not significantly change the structure of the soil microbial community (He et al., 2017). To further explore these dynamics, the effects of plant genotype on the bacterial communities in maize (Aira et al., 2010), soybean (Sugiyama et al., 2014), rice (Shenton et al., 2016), common bean (Mendes et al., 2018a,b) and lima bean (Sousa et al., 2020) have been well studied. In addition, the mulberry and alfalfa intercropping system changed the soil physical and chemical properties and soil bacterial community (Zhang et al., 2018). However, little is known about the relationships between perennial alfalfa cultivars, rhizosphere soil properties, and soil bacteria. Plant–microbe–soil feedback can influence plant growth and the nutrient cycle, which play a fundamental role in plant community establishment and success (Baxendale et al., 2014). These interactions contribute to the nutrient acquisition of alfalfa and substantially increase the fitness of the host and of various soil properties.

Therefore, we determined the bacterial community composition as well as physiochemical properties and enzyme activities in rhizosphere soils of 12 perennial alfalfa cultivars. The goal of this experiment was to assess the effects of different cultivars on soil bacterial community diversity, composition, and networks. Finally, compared these effects to the relative influence of nitrogen content in long-term plant-soil histories. Differences in microbial enrichment in the alfalfa cultivars were observed based on the identification of the rhizosphere microbial community (Ma et al., 2020). In addition, the physiochemical properties of the rhizosphere soil were determined to explore the relationship between the microbial community composition and diversity. Our results illustrate how plant and soil microbes play a role in nitrogen content and which of these plant-mediated changes are most important in response to soil nitrogen content.

## Materials and methods

### Sample site and experimental design

The study was conducted at the experimental station of Northwest A&F University, located in Yangling Shaanxi (34°16′ N, 108°4′ E). The area has a warm temperate monsoon semi-humid climate with a mean annual temperature of 12.9°C and total annual precipitation of approximately 600 mm, and approximately 60% of the precipitation occurs between July and September. The soil type was Lou (Eum-Orthic Anthrosol; Zhong et al., 2020).

The planting area was 60 m × 18 m, dozens of alfalfa cultivars have been cultivated since 2012 under a green harvest management, between the different cultivars were separated by 1 m ridges. Before planting, the plots were plowed and homogenized to reduce soil variability. Clean cultivation, compound fertilizer (dissolved in water) and hand weeding are conventional practices for alfalfa cultivation in this region. Alfalfa sampled in this study was mowed three times per year and did not show evidence of pest attack, disease, or nutritional deficiency. After 20 days of cutting in July 2020, the alfalfa entered the budding stage (Fan et al., 2018). Twelve alfalfa cultivars samples (Table 1) were selected from dozens of alfalfa cultivars in the same growth stage, refers to budding stage.

**TABLE 1:** The twelve cultivars of alfalfa for this study.

No.	Cultivars	Abbreviations	Source of country
1	Victorian	Vt	Canada
2	Serbia	Sb	Serbia
3	Sardi 10	Sd	Australia
4	Mudi	Md	America
5	Forerunner	Fr	France
6	Muzi 401	Mz	China
7	Rambler	Rb	America
8	NS05	Ns	China
9	MF4020	Mf	America
10	NS-ZMS-V	Zm	China
11	WL363HQ	Ws	America
12	WL343HQ	Wf	America

In this study, the plots had 18 m^2^ of each alfalfa cultivar, being 6 m^2^ the usable area for soil sampling. Before soil sampling, we used the five-point sampling method, as previously described (Navarrete et al., 2015). Five samples were obtained at five cardinal points around the root of alfalfa, and collection was carried out by digging an area of 10 × 10 × 40 cm (length × width × depth) after removing the litter layer. A soil drill was used to obtain mixed samples of soil and roots, and fine roots were picked out. Soil that adhered to the roots was collected and mixed to form a composite sample for each plot. Five plants (biological replicate) were selected randomly from the plots of each cultivar of alfalfa, generating a total of 60 samples (12 alfalfa cultivars × five repeats). Before collecting the next soil sample, the residue on the shovel was wiped with sterile paper, and the sample was disinfected to avoid contamination between treatments. All samples were passed through a sterile 2 mm sieve before use. All soil samples were obtained and stored at –80°C for further analysis.

### 16S rRNA gene sample preparation, sequencing, and analysis

In total, 60 samples from the alfalfa rhizosphere were used for lumina-based 16S rRNA gene sequencing. Microbial DNA was extracted using the DNeasy 96 PowerSoil Pro QIAcube HT Kit (QIAGEN, Germany). The V3-V4 hypervariable region of the bacterial 16S rRNA gene was amplified using the primers 338F (ACTCCTACGGGAGGCAGCAG) and 806R (GGACTACHVGGGTWTCTAAT; Xu et al., 2016). PCR was performed as follows: 95°C for 3 min; 30 cycles of 95°C for 30 s, 55°C for 30 s, and 72°C for 45 s; and 72°C for 10 min. 10°C until it was halted by the user. The products were extracted from 2% agarose gels and purified using the AxyPrep DNA Gel Extraction Kit (Axygen Biosciences, Union City, CA, United States). The amplicons were pooled in equimolar concentrations and paired-end sequenced (2 × 300) on an Illumina MiSeq platform (Illumina, San Diego, CA, United States) according to standard protocols at Majorbio Bio-Pharm Technology Co., Ltd. (Shanghai, China).

### Growth and nutrition index of alfalfa

During harvesting, the total fresh weight of each plant (biomass of the aerial part of the plant), called aboveground biomass (BM), was collected to account for plant tissue nutrient characteristics. The crude protein content (CP) of alfalfa leaves was determined by H_2_SO_4_-H_2_O_2_ digestion using Kjeldahl nitrogen (Mulvaney et al., 1992).

### Soil physiochemical properties

Soil characteristics such as pH, EC, and total phosphorus (TP), available phosphorus (AP), soil organic carbon (SOC), total nitrogen (TN), ammonium nitrogen (AN) and nitrate nitrogen (NT) were determined following standard methods (Jing et al., 2015). In brief, soil pH and EC were determined using a 5:1 water-to-soil ratio. TP was determined using HClO_4_-H_2_SO_4_ digestion molybdenum antimony anticolorimetry. AP was determined by Na_2_CO_3_ extraction using molybdenum–antimony inverse colorimetry. SOC was determined using the KCr_2_O_7_-H_2_SO_4_ external heating method. TN was determined using the semi-micro Kjeldahl method. AN and nitrate nitrogen (NT) were determined by 2 mol/L KCl extraction continuous flow analyzer (Wang et al., 2014).

### Soil microbial biomass and extracellular enzyme activities

Soil microbial biomass carbon (MBC) and nitrogen (MBN) were determined using the chloroform fumigation extraction method followed by K_2_SO_4_ soil extraction (Vance et al., 1987), and a total organic carbon analyzer was used for analysis. Urease activity (URE) was determined by sodium phenolate sodium hypochlorite colorimetry using urea as the substrate. Leucine peptidase (LAP) and β-1,4-*n*-acetylglucosaminidase (NAG) activities were measured using a modified microplate fluorescence method (Saiya-Cork et al., 2002), and soil enzyme activity [nmol/(g⋅h)] was expressed as the matrix conversion rate of 1 g sample per unit time. Nitrogen fixation rates (NAT) were measured using acetylene conversion to ethylene (ARA), as previously described (Tao et al., 2019). The C_2_H_4_ concentration of each gas sample was measured using a gas chromatograph (Trace GC UL tra, Thermo Fisher, United States). ARA was determined using gas chromatography (SRI Instruments).

### Data analyses

Data were tested for normality (Kolmogorov–Smirnov test) and homoscedasticity (Levene’s test). One-way analysis of variance (ANOVA) followed by Tukey’s HSD test (*P* < 0.05) was used to evaluate differences in plant crude protein content, aboveground biomass, soil properties, microbial diversity, and relative abundance of bacteria among the 12 cultivars. Tukey *post hoc* comparisons were used for all univariate analyses using SPSS v 25.0 (SPSS: IBM Corp). We performed redundancy analysis (RDA) to identify the effects of plants and soil on microbial community composition (Oksanen et al., 2013). SPSS 25.0 software was used to perform a Spearman correlation analysis of soil properties and phylum-level bacterial composition. The Spearman correlation coefficients of the top 50 most abundant bacterial genera, soil properties, and plant characteristics were calculated and displayed on the heat map. The relationship between microbial community composition, diversity attributes, and soil nitrogen was assessed using the corrplot package in R.

Network analysis was performed on the alfalfa rhizosphere microorganisms and soil properties based on strong and significant correlations (non-parametric Spearman’s correlation, *P* < 0.01, and absolute value of *r* > 0.6). Low-abundance genera were eliminated from the taxonomy table when they comprised less than 0.01% of the total relative abundance across all samples before network analysis. Statistical analysis of the network was carried out in the R environment, and correlation networks were visualized using the Gephi software (Bastian et al., 2009).

Structural equation modeling (SEM; Grace et al., 2012) was used to better understand the relationship between plant and soil variables and their impact on total nitrogen. Structural equation modeling was performed using the online software PLS (Ringle et al., 2015) based on the principle of the partial least squares path model. The priori SEM was originally constructed based on the bivariate correlation of plant and soil variables, in which direct and indirect pathways were considered. The normality of variables, linearity between variables, and non-multicollinearity between variables were verified before operating the model. To obtain the most concise model, insignificant and uninformative weak paths were removed from the final model. The maximum likelihood chi-square test was used (χ^2^, *P* > 0.05), Tucker-Lewis index (TLI ≥ 0.90), comparative fit index (CFI ≥ 0.90), the goodness of fit (GFI ≥ 0.90), and mean square error of root evaluation model fitting approximation (RMSEA < 0.10). The significance level was set at *P* < 0.05.

## Results

### Crude protein and biomass changes in alfalfa cultivars

**FIGURE 1:**
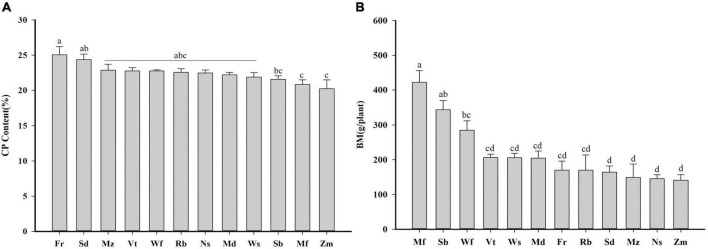
Average values of twelve alfalfa cultivars: **(A)** Crude protein content of alfalfa above ground (CP), **(B)** Aboveground biomass of alfalfa (BM, g plant^– 1^), fresh weight per plant. One-way analysis of variance, followed by Tukey’s honestly significant difference test was performed (*P* < 0.05). Different lowercase letters indicate significant differences among mean values in different cultivars. The alfalfa cultivars from high mean value to low mean value are shown from left to right.

Significant differences were found among alfalfa cultivars for crude protein and biomass (fresh weight per plant; Figure 1). The crude protein content ranged between 20.20 and 25.05 g kg^–1^ (Figure 1A), and the aboveground biomass ranged from 141.03 to 422.08 g plant^–1^ (Figure 1B). Crude protein of plants was highest in Fr, and comparatively lower in Zm and Mf compared to the other cultivars (*P* < *0.05*). The cultivar producing largest biomass was Mf; and, Zm, Ns, Mz, and Sd were much lower than those of the other cultivars.

### Differing rhizosphere soil properties of alfalfa cultivars

Soil physiochemical characteristics were also significantly affected by cultivars. The pH values were moderately alkaline and generally remained consistent among cultivars; with the highest and lowest pH values found in Ns (8.24) and Mf (8.03), respectively (Table 2). The SOC contents were highest in Mf (11.86 g kg^–1^) and lowest in Ns (8.70 g kg^–1^), and were significantly different (*P* < *0.05*; Table 2). The AP and TP contents showed similar trends, except for Sb and Sd, which had higher TP and lower in AP levels compared to other cultivars for all cultivars, except Sb and Sd, which had higher TP and lower AP levels compared to other cultivars (Table 2). In addition, significant changes in EC were found in some cultivars (Table 2).

**TABLE 2:** The rhizosphere soil properties in different alfalfa cultivars.

Alfalfa cultivars	pH	EC (us/cm)	SOC (g/kg)	TP (g/kg)	AP (mg/kg)
Vt	8.16 ± 0.04^abcd^	128.62 ± 1.04^bcde^	10.13 ± 0.53^abcde^	1.60 ± 0.01^a^	6.54 ± 0.51^a^
Sb	8.15 ± 0.02^abcd^	133.70 ± 1.39^abc^	10.25 ± 0.49^abcde^	1.60 ± 0.01^a^	5.12 ± 0.25^abc^
Sd	8.21 ± 0.01^ab^	116.82 ± 1.58^e^	8.91 ± 0.41^de^	1.55 ± 0.00^abc^	4.28 ± 0.26^c^
Md	8.09 ± 0.03^cde^	121.36 ± 1.68^de^	9.61 ± 0.33^bcde^	1.44 ± 0.04^cd^	5.26 ± 0.17^abc^
Fr	8.09 ± 0.01^cde^	141.20 ± 2.61^a^	11.42 ± 0.01^ab^	1.44 ± 0.03^cd^	4.92 ± 0.64^abc^
Mz	8.18 ± 0.01^abc^	126.38 ± 2.30^bcde^	9.40 ± 0.26^cde^	1.41 ± 0.02^de^	5.12 ± 0.17^abc^
Rb	8.12 ± 0.01^bcde^	122.02 ± 2.57^cde^	10.00 ± 0.49^abcde^	1.59 ± 0.01^ab^	5.14 ± 0.50^abc^
Ns	8.24 ± 0.01^a^	124.00 ± 1.94^bcde^	8.70 ± 0.21^e^	1.31 ± 0.03^e^	4.60 ± 0.29^bc^
Mf	8.03 ± 0.02^e^	131.44 ± 3.17^abcd^	11.86 ± 0.45^a^	1.46 ± 0.04^bcd^	6.34 ± 0.37^a^
Zm	8.09 ± 0.01^cde^	128.50 ± 4.06^bcde^	10.33 ± 0.64^abcde^	1.53 ± 0.04^abcd^	6.06 ± 0.24^ab^
Ws	8.04 ± 0.00^e^	134.10 ± 2.96^ab^	10.79 ± 0.42^abcd^	1.55 ± 0.04^abc^	5.56 ± 0.15^abc^
Wf	8.08 ± 0.02^de^	128.74 ± 2.83^bcde^	11.22 ± 0.36^abc^	1.54 ± 0.01^abcd^	6.44 ± 0.19^a^

Soil properties (pH value, soil electrical conductivity, soil organic carbon, soil total phosphorus, and soil available phosphorus) among different alfalfa cultivars. Different lowercase letters indicate significant differences (*P* < 0.05) among mean values in different cultivars. Values are means ± standard error (n = 5).

### Nitrogen content and microbial biomass in plant rhizosphere soil

**FIGURE 2:**
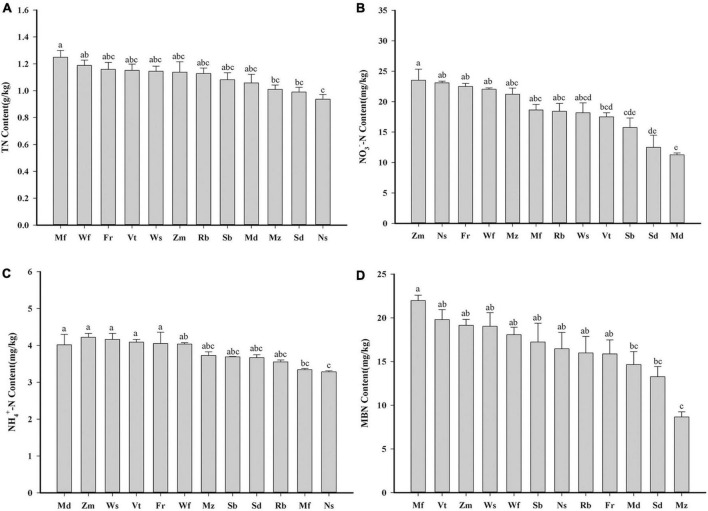
Average values of nitrogen in soil: **(A)** total nitrogen (TN, g kg*^–^*^1^), **(B)** nitrate nitrogen (NT, mg kg*^–^*^1^), **(C)** ammonium nitrogen (AN, mg kg*^–^*^1^) and **(D)** microbial biomass nitrogen (MBN, mg kg*^–^*^1^) of different alfalfa cultivars. Data were obtained from five biological replicates. One-way analysis of variance, followed by Tukey’s honestly significant difference test was performed (*P* < 0.05). Statistical classes sharing a lowercase letter are not significantly different. The alfalfa cultivars from high mean value to low mean value are shown from left to right.

The total nitrogen content of all soil samples ranged between 0.94 and 1.25 g kg^–1^ (Figure 2A). TN contents of Mf were significantly higher than Mz, Sd and Ns (*P* < 0.05). The soil NO_3_^–^-N (NT) content varied widely among different cultivars, ranged between 11.28 and 23.53 mg kg^–1^ (Figure 2B). NH_4_^+^-N (AN) content ranged between 3.29 and 4.22 mg kg^–1^ (Figure 2C). Md had the highest AN content and lowest NT content. Mf had the highest MBN content (21.98 mg kg^–1^; Figure 2D). Mz showed the lowest content of MBN and MBC compared to the other cultivars (Figure 2D and Supplementary Figure 1D).

### Taxonomic classification, microbial rhizosphere community diversity, and community composition

**FIGURE 3:**
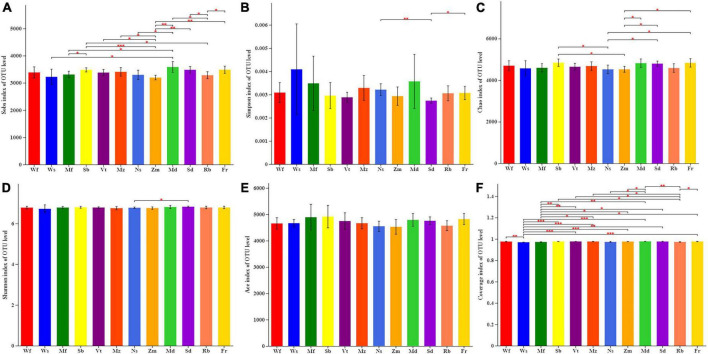
Average values of twelve alfalfa cultivars on bacterial alpha-diversity of OTU: **(A)** Sobs index, **(B)** Simpson diversity, **(C)** Chao richness, **(D)** Shannon diversity, **(E)** Ace richness, **(F)** Coverage index. *0.01 < *P* ≤ 0.05, ^**^0.001 < *P* ≤ 0.01, ^***^P ≤ 0.001.

The number of bacterial sequences varied from 38,220 to 73,626 per sample (mean: 55,209), and the average read length of the bacteria was 418 bp. For downstream analysis of the bacterial sequences, the datasets were rarefied to 3,312,565 valid sequences. Among the valid sequences obtained from alfalfa soil samples, an average of 7592 OTUs were obtained. The Sobs index showed that the total number of OTUs in addition to Wf was significantly different from that of other alfalfa cultivars (Figure 3A); among them, the total number of OTUs in Md was the highest and that in Zm was the lowest (Supplementary Figure 3A). There were significant differences between the two cultivars Mf and Sd. In terms of Simpson index (Figure 3B), only Ns and Fr were significantly higher than Sd. The Chao1 index was significantly higher in Sb, Fr, and Sd than that in Ns and Zm. According to the Shannon index, only Ns and Sd differed significantly (Figure 3C). No significant difference was found in the ACE index among the 12 alfalfa cultivars in this study (Figure 3D).

**FIGURE 4:**
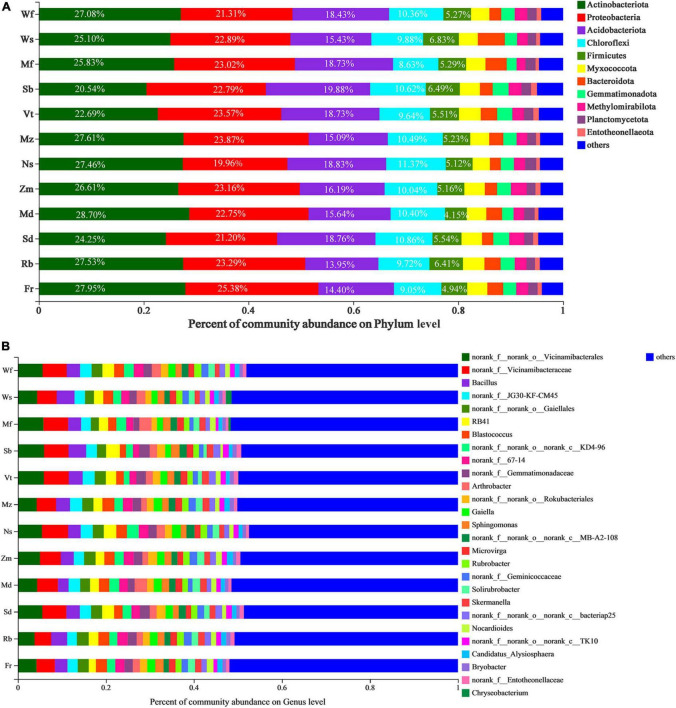
Taxonomic profiles of twelve alfalfa cultivars at the phylum **(A)** and genus **(B)** level, with higher relative richness are shown in the form of percentage in the figure, the remainder were combined and lumped in a category designated as “Other.” Different colors represent different species, and the length of each bar represents the proportion contributed by each species.

From all alfalfa cultivars in this study, we identified ten bacterial phyla: Actinobacteria (25.91%), Proteobacteria (22.79%), Acidobacteria (17.03%), Chloroflexi (10.10%), Firmicutes (5.46%), Myxococcota (3.77%), Bacteroidetes (2.91%), Gemmatimonadota (2.58%), Methylomirabilota (2.30%), Planctomycetota (1.78%) and others (5.35%; Supplementary Figure 2). For each alfalfa cultivar, the percentage of the total reads corresponding to each phylum (Figure 4A). Significant differences were observed in Actinobacteriota, Gemmatimonadota and Entotheonellaeota in bacterial phyla of different alfalfa cultivars (Supplementary Figure 3). At a genus level (Figure 4B), the most abundant genus in the rhizosphere soil of all alfalfa cultivars was an unknown genus, followed by Vicinamibacterales and Vicinamibacteraceae which belong to the Acidobacteriota phylum.

### Correlations between physiochemical properties, microbial biomass, bacterial community composition and diversity

**FIGURE 5:**
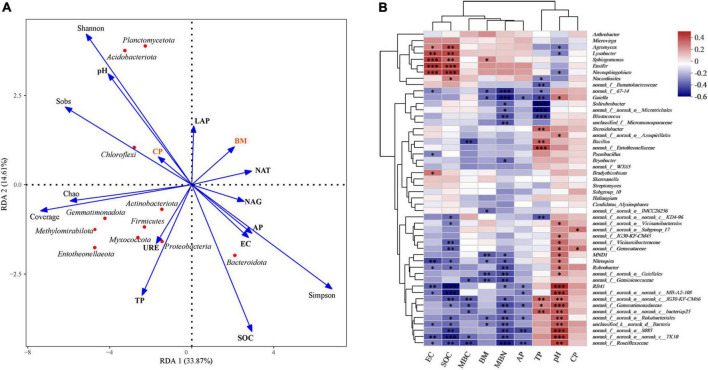
**(A)** Redundancy analysis (RDA) shows relationships between plant (BM and CP), soil physiochemical characteristics (pH, SOC, EC, AP, TP), microbial communities. The values of axes 1 and 2 are the percentages explained by the corresponding axis. Analysis of the level of contribution of soil, plant characteristics and bacterial diversity to changes in bacterial communities. **(B)** Correlation heat map of the top fifty genera and soil properties, the *R* value is shown in different colors in the figure. *0.01 < *P* ≤ 0.05, ^**^0.001 < *P* ≤ 0.01, ^***^P ≤ 0.001.

RDA (Figure 5A) was used to quantify the variations in plant and soil properties and its relationships with soil microbial community composition and structure. It was revealed that soil properties (pH, SOC, and TP) and microbial diversity were more influential on soil bacterial communities than plant crude protein and biomass. RDA supported the driving forces of soil properties on bacterial communities at the phylum level (Figure 5A) and showed a correlation between the two variables. The pH value was positively correlated with Acidobacteria, Chloroflexi, and Gemmatimonadota but negatively correlated with Bacteroidetes. TP content was positively correlated with Firmicutes and Gemmatimonadota but negatively correlated with Actinobacteria; however, available P content was negatively correlated with Gemmatimonadota and positively correlated with Firmicutes. SOC was negatively correlated with Acidobacteria, Chloroflexi, and Gemmatimonadota but positively correlated with Bacteroidota (Supplementary Table 3).

Spearman correlation of plant characteristics, soil properties, and microbial biomass in genus taxa, and the results are displayed as heat maps (Figure 5B). *Agromyces*, *Lysobacter*, *Sphingomonas*, *Ensifer* and *Novosphingobium* showed an extremely positive correlation with EC and SOC, in which *Ensifer* belongs to *Rhizobiales*. *Nocardioides* showed a significant positive correlation with SOC and *Bradyrhizobium* belongs to *Rhizobiales*. In our study, we found that most bacteria, such as *Gemmatimonadaceae*, *RB41*, *Rokubacteriales*, *Gaiella*, *Vicinamibacteraceae*, *Rubrobacter*, and *JG30-KF-CM45*, demonstrated an extremely significant positive correlation with pH, whereas *Vicinamibacteraceae*, *RB41*, *Gemmatimonadaceae*, and *Rubrobacter* were significantly negative correlated with SOC. MBN was significantly negatively correlated with *Roseiflexaceae*, *Gemmatimonadaceae*, *Gaiella*, *Geminicoccaceae*, and *Rokubacteriales* (Figure 5B).

### Correlations between soil nitrogen content, bacterial community composition, and diversity

**FIGURE 6:**
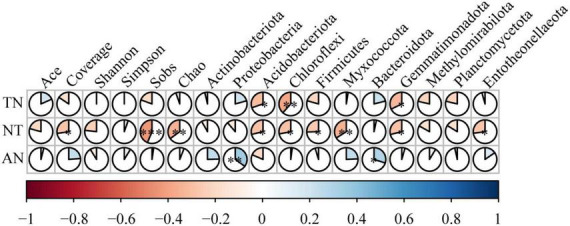
Spearman correlation coefficients between the soil nitrogen content, soil microbial diversity and soil microbial communities. *0.01 < *P* ≤ 0.05, ^**^0.001 < *P* ≤ 0.01, ^***^P ≤ 0.001.

TN and NT were significantly negatively correlated with Acidobacteria, Chloroflexi, and Gemmatimonadota. NT was significantly negatively correlated with Firmicutes, Myxococcota, and Entotheonellaeota. In contrast, AN was significantly positively correlated with Proteobacteria and Bacteroidetes. The bacterial alpha diversity indices indicated no significant effects on TN and AN content (Figure 6). The exception was NT, which showed the same results as the cultivars in the relationship with bacterial diversity. The major factor affecting nitrogen content is the dominant bacterial community in terms of bacterial diversity, with differences in total and inorganic nitrogen.

### Soil bacterial community responses to nitrogen content in alfalfa

**FIGURE 7:**
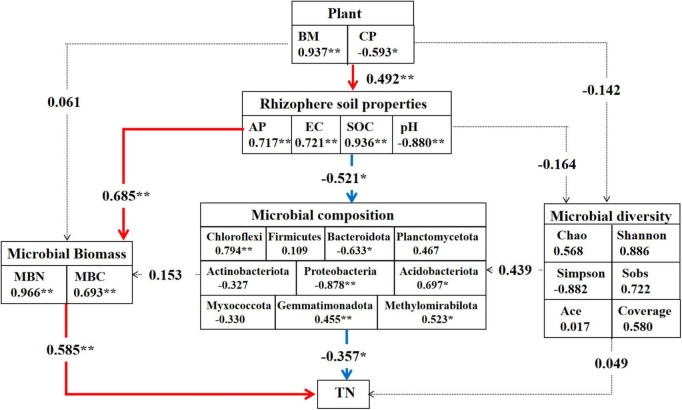
Partial least squares path models (PLS-PM) of the drivers of soil nitrogen content. Path analysis results for direct and indirect effects of the plant, rhizosphere soil properties, microbial biomass and composition on soil nitrogen content. The asterisks denote statistically significant differences among different alfalfa cultivars (*P < 0.05, **P < 0.01). Numbers on arrows are path coefficients indicating a positive (positive number) in red or negative effect in green (negative number).

Based on partial least squares path models, we established that plants, soil, and microbiota were significantly associated with both direct and indirect effects on the response to soil nitrogen through our hypothesized pathways, involving the plant index, soil properties, microbial biomass, microbial composition, and diversity. The results revealed that the rhizosphere soil properties were positively associated with the plant index (Figure 7). Moreover, most soil properties had a significantly positive effect on microbial biomass and composition, including SOC, EC, and AP, except for the pH value. Soil microbial biomass had a direct positive correlation with the soil total nitrogen content. Interestingly, most microbial communities were shown to have a direct negative effect on soil nitrogen content, including Acidobacteria, Chloroflexi, Gemmatimonadota, and Methylomirabilota, whereas Proteobacteria and Bacteroidetes had direct positive effects on soil nitrogen content. Notably, soil microbial diversity appeared to have weaker correlations with plant and soil nitrogen content than with soil microbial biomass and composition.

## Discussion

### Effects of plant cultivars on soil properties

In natural ecosystems, plants grow in their native soil, and the characteristics of the soil near plant roots are modified by a range of plant growth processes, which in turn affect the rhizosphere microbiota (Philippot et al., 2013). Plant genotypes have a significant effect on the phylogenetic structure of root-associated microbiota (Hamonts et al., 2018). Under the same environmental conditions and soil, plant genotype is the main factor affecting the structure and function of rhizosphere microbiome (Zhang et al., 2017). By changing the properties of the surrounding soil, plants can modify their growth environments (Hu et al., 2018). Based on previous research, we hypothesized that differences in alfalfa cultivars induce changes in soil properties, which can significantly affect the soil nitrogen content and structure of rhizosphere microbial communities. We examined 12 different alfalfa cultivars from the same area with the same growth period, cutting method, cutting time, and similar initial soil. Some differences were observed in the crude protein and biomass per plant among the different alfalfa cultivars (Figure 1), which might be related to the productivity of plants, since it depends on the ability to acquisition of water and nutrients from the soil. In other words, the chemical characteristics of a plant species affect the properties of the surrounding soil (Song et al., 2019). In addition, Plants can directly alter the soil microbiome by secreting and releasing bioactive molecules and metabolites (Pang et al., 2021). Root exudates may differ between plant species, different growth stages, and even between the same plant species (Micallef et al., 2009; Li et al., 2013; Dietz et al., 2019), therefore, root exudates from the twelve alfalfa cultivars might have led to changes in soil physiochemical properties (Zhang et al., 2020), and most likely contributed to the effects on soil microbial activity and biomass (Gomez-Sagasti et al., 2021).

### Effects of rhizosphere soil properties on soil microorganisms

Plant–soil feedback is associated with maintaining or changing the soil community, and soil rhizosphere bacteria can be influenced by both soil physiochemical properties and enzyme activities (Bani et al., 2018). We illustrated that, among specific perennial alfalfa cultivars, there were differences in changing soil properties and the recruitment of microorganisms. Hence, this is a fact that plants indirectly affect nutrient cycling by affecting the activities of soil decomposers and the decomposition of organic matter (Henneron et al., 2020). The critical roles of pH and SOC in shaping the bacterial community structure are characterized well (Figure 5A; Ling et al., 2016; Qu et al., 2020). The pH value can be increased or decreased by plant roots owing to the release or absorption of ions (Hinsinger et al., 2009), which gives rise to differences in soil pH values between species and even cultivars in the plant rhizosphere (Table 2). All these changes indicates that although the soil was uniform before sowing, but after years of growth of different cultivars has caused significant alterations in soil properties. Soil pH has significant effects on alfalfa rhizome microecosystems (Xiao et al., 2017), which mainly causes different microorganisms to favor different optimal pH values. The strong correlation between soil pH and microbial distribution could be due to the relatively narrow growth tolerance exhibited by most bacterial taxa (Xue et al., 2017). Soil organic carbon act as the soil carbon pool, provide rich carbon sources for the activities of microorganisms (Gan et al., 2013). This may coincide with the results that the rhizosphere soil properties directly affect the soil microbial biomass (Figure 7). RDA showed that soil properties (pH, SOC, and EC) and enzyme activity (NAT and URE) were the primary factors influencing bacterial community composition (Figure 5A). Meanwhile, soil microbial biomass is the source and sink of plant nutrients and actively participates in the nutrient cycle, representing the active part of the soil nutrients (Jia et al., 2017; Henneron et al., 2020). Taken together, these results indicate that soil properties, especially pH and SOC play a vital role in determining the bacterial community composition in perennial alfalfa.

### Microbial biomass and community can directly regulate soil nitrogen content

The composition of the soil microbial community is considered an important component of the plant–soil feedback process (Bever et al., 2012). Soil microbial biomass nitrogen content is a reflection of soil microorganisms on nitrogen mineralization and fixation (Li et al., 2018) and further affects the content of total nitrogen and inorganic nitrogen in the soil. Concurrent with changes in MBN and MBC, the SEM model revealed a positive correlation with TN content (Figure 7). Our research indicates that soil bacterial diversity has a minimal effect on soil nitrogen (Figures 6, 7), possibly because all cultivars are closely related and belong to the legume family. Microbial species interactions are crucial to the structure and dynamics of soil bacterial communities (Chai et al., 2019). Thus, we further used co-occurrence network analysis to explore the complexity of the connections within the rhizosphere microbiomes of the different perennial alfalfa cultivars. First, the network complexity of the root system has a profound impact on nitrogen content. The higher content of TN in Mf indicates that the overall impact of the root network on the crude protein was much greater than the soil properties (Supplementary Figure 4). In Ws, the dominant bacteria belonging to Myxococcota, Chloroflexi, Entotheoellaeota, Bacteroides, and Acidobacterita had lower network complexity and accounted for a lower proportion of the composition of the total bacterial community. In Ns and Fr, the dominant bacteria belonged to Proteobacteria, Actinobacteria, Chloroflexi, and Myxococcota, which play an important role in the composition of the whole bacterial community. Nevertheless, the TN content of Md was the lowest, which demonstrated stronger correlations in this ecological network, possibly because the number of negative correlations was the highest among the edges compared with the other cultivars, implying that inter-competitive exclusion occurred within the bacterial community (Feng et al., 2017). The results showed that the dominant microbial population plays a dominant role in the rhizosphere. The most abundant phyla studied in the co-occurrence network were Acidobacteria and Proteobacteria, indicating that these generalists are adapted to a variety of environments (Xue et al., 2017). Proteobacteria are capable of inducing nitrogen fixation in symbiosis with plants (Emmert and Handelsman, 1999). The high abundance of Proteobacteria confirms that this phylum seems to benefit from the characteristics of leguminous plants (Sousa et al., 2020) and exhibited the variation by cultivar in our study (Figure 6). Plants can alter their rhizosphere microbiomes by influencing nutrient availability (Bell et al., 2015). These results indicate that rhizosphere microbial community composition, rather than diversity, plays a dominant role in affecting soil nitrogen. Similar to our findings (Figure 6), different biological and chemical drivers of each lima bean genotype influence the bacterial structure (Sousa et al., 2020). In this study, a significant relationship between bacterial community structure and soil total nitrogen content was also observed, indicating that variables associated with bacterial community composition may be crucial determinants of total nitrogen content (Figure 7).

Our results suggest that the differences in perennial alfalfa cultivars influenced soil physiochemical properties and further influenced soil microbial composition and biomass (Figure 7). The ability of plants to establish beneficial rhizosphere bacterial communities may be an important aspect of plant adaptability and should be considered an intrinsic plant trait that may be selected (Bell et al., 2015). The community structure of a certain cultivar in the rhizosphere showed a relatively low abundance, which may be due to its low ability to change the community structure of rhizosphere bacteria. The dynamics of soil microbial communities are likely determined by plants and the mediation of soil nutrient availability (Cabugao et al., 2017). We postulated that the difference in alfalfa may have unintentionally co-selected, affecting the recruitment of beneficial cultivar-specific microbiota, and finally affecting the soil nitrogen content. Further investigations are required to reveal the mechanisms underlying this specific microbial recruitment process.

## Conclusion

This study reported differences in the rhizosphere soil microbial community and soil nitrogen content among different perennial alfalfa cultivars. According to the PLS-PM analysis, changing soil properties indirectly affected microbial biomass and community composition. Soil pH and SOC had a strong correlation with bacterial composition and diversity. Microbial biomass and soil total nitrogen are positively correlated, and some key microbial species play a major role. This study provides insight into the distributional patterns and drivers of soil total nitrogen in rhizosphere soil of perennial alfalfa and improves our understanding of the relationship between alfalfa, physiochemical properties and microbial.

## Data availability statement

The original contributions presented in this study are publicly available. This data can be found here: NCBI; PRJNA812793.

## Author contributions

YA and PY contributed for experimental design, sampling, lab analysis, interpretation and analysis of data, and writing the manuscript. HS, WZ, and ZY contributed in experimental design, interpretation of data, and manuscript revisions. SL and YS contributed in interpretation of data. RY contributed in lab analysis. TH corrected the draft. All authors contributed to the article and approved the submitted version.
