# Supplementary material for: Distinct rhizosphere soil responses to nitrogen in relation to microbial biomass and community composition at initial flowering stages of alfalfa cultivars
Source: Front Plant Sci. 2022 Aug 24;13:938865. doi: 10.3389/fpls.2022.938865 (PMC9449485; doi:10.3389/fpls.2022.938865)
Supplement: Supplementary file 1 [file Data_Sheet_1.doc]

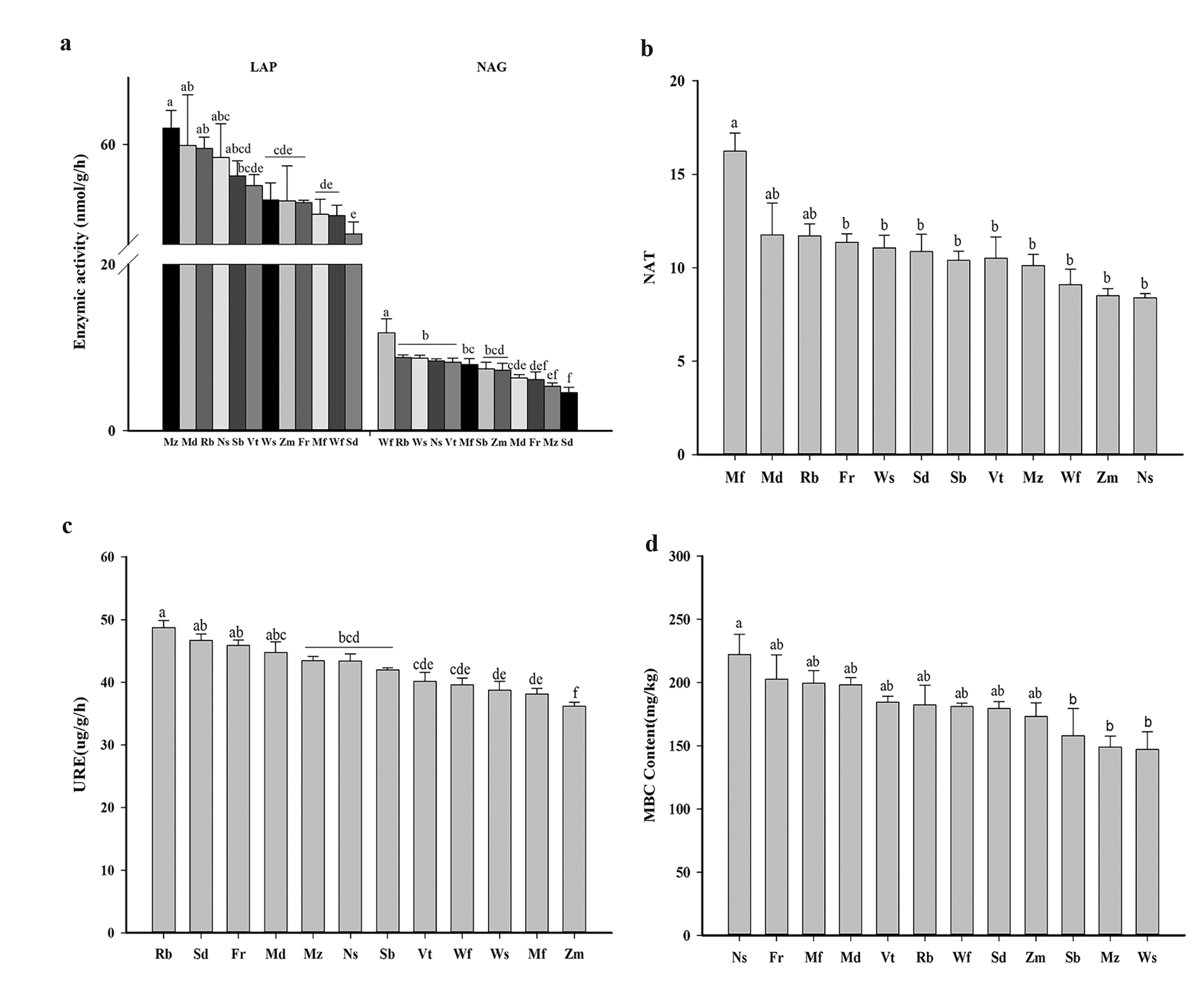


Figure S1. Average values of enzyme activities: a leucine aminopeptidase (LAP, nmol g^-1^h^-1^), β-1,4- N -acetylglucosaminidase (NAG, nmol g^-1^h^-1^), b Nitrogenase (NAT, μ mol g^-1^h^-1^), c urease (URE, ug g^-1^h^-1^) and d microbial biomass carbon (MBC, mg^-1^kg) of different cultivars in soil. One‐way analysis of variance, followed by Tukey’s honestly significant difference test was performed (P<0.05). Different lowercase letters indicate significant differences among the mean values of different cultivars. The alfalfa cultivar from high mean value to low mean value is shown from left to right.


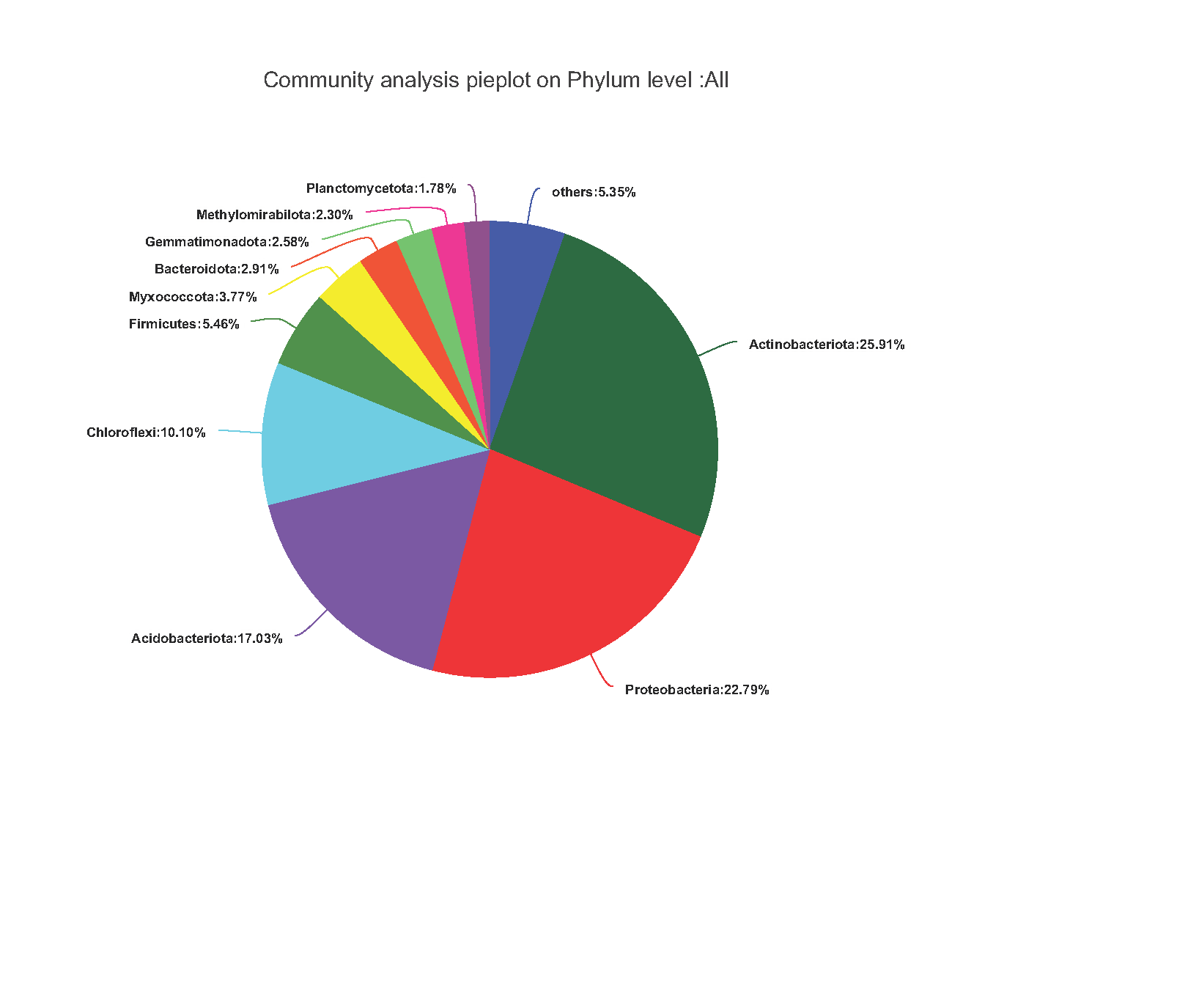


Figure S2. All the samples of community analysis pieplot on phylum.


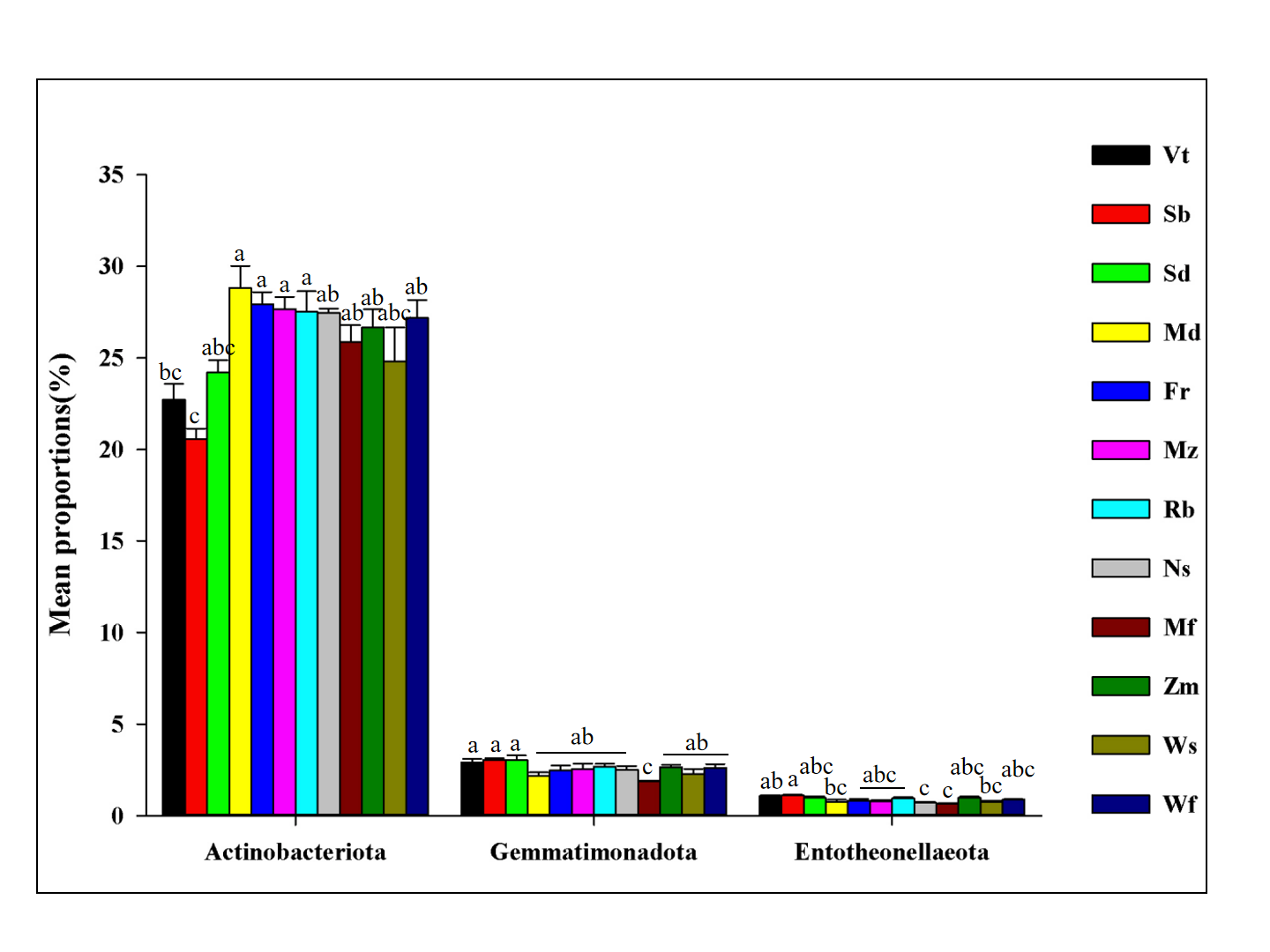


Figure S3. Statistical comparison of the relative abundance of rhizosphere soil microbiota among the twelve alfalfa cultivars. One‐way analysis of variance, followed by Tukey’s honestly significant difference test was performed (P<0.05). Different lowercase letters indicate significant differences among the mean values of different cultivars.


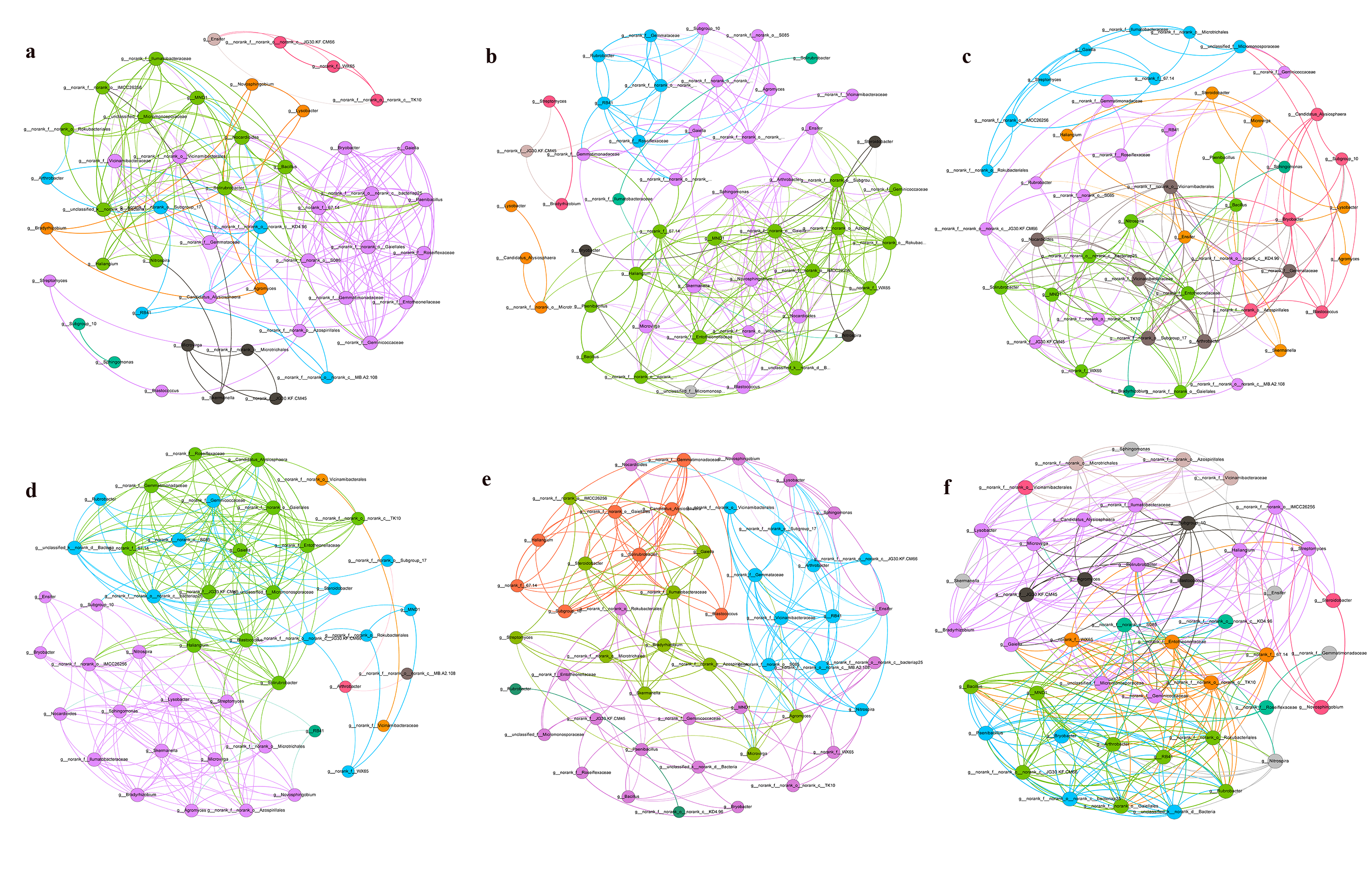


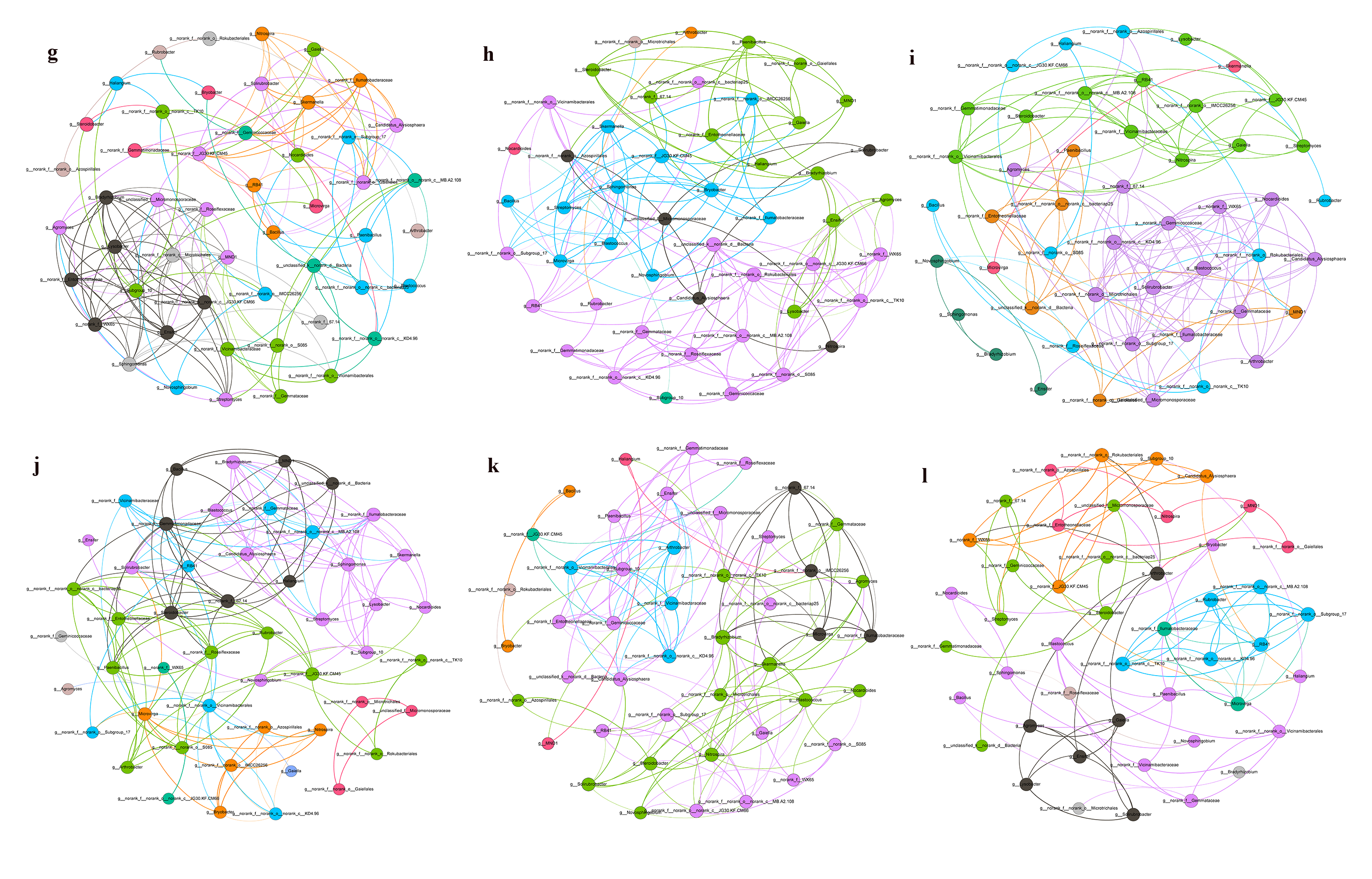


Figure S4. The networks of the co-occurring bacterial genus in the rhizosphere soil of twelve alfalfa cultivars: a Fr, b Md, c Mz, d Ns, e Rb, f Ws, g Wf, h Sb, i Sd, j Vt, k Zm and l Mf based on correlation analysis. A connection stands for a strong (Spearman's r>0.6) and significant (P<0.01) correlation. The co-occurring networks are colored by the modularity class.

Table S2 Topological properties of co-occurring bacterial networks obtained among the rhizosphere of twelve alfalfa groups, and their corresponding random networks.

|  | Vt | Sb | Sd | Md | Fr | Mz | Rb | Ns | Mf | Zm | Ws | Wf |
| --- | --- | --- | --- | --- | --- | --- | --- | --- | --- | --- | --- | --- |
| Number of nodes | 50 | 49 | 48 | 50 | 48 | 48 | 50 | 46 | 46 | 46 | 46 | 50 |
| Number of edges | 161 | 160 | 144 | 230 | 205 | 154 | 180 | 229 | 96 | 149 | 273 | 162 |
| Number of positive correlations | 173 | 172 | 190 | 182 | 207 | 145 | 197 | 274 | 126 | 128 | 311 | 89 |
| Number of negative correlations | 38 | 38 | 4 | 98 | 48 | 59 | 33 | 5 | 60 | 71 | 12 | 16 |
| Average path length (APL) | 3.765 | 3.526 | 3.802 | 2.895 | 2.684 | 4.178 | 3.657 | 3.051 | 4.391 | 3.368 | 2.753 | 3.441 |
| Graph Density | 0.131 | 0.136 | 0.128 | 0.188 | 0.182 | 0.137 | 0.147 | 0.221 | 0.093 | 0.144 | 0.264 | 0.132 |
| Network diameter | 8 | 10 | 11 | 8 | 9 | 12 | 10 | 10 | 12 | 8 | 7 | 8 |
| Average clustering coefficient (avgCC) | 0.71 | 0.634 | 0.582 | 0.705 | 0.754 | 0.596 | 0.558 | 0.686 | 0.633 | 0.557 | 0.66 | 0.561 |
| Average degree (avgK) | 6.44 | 6.531 | 6 | 9.2 | 8.542 | 6.417 | 7.2 | 9.957 | 4.174 | 6.478 | 11.87 | 6.48 |
| Number of modules | 10 | 8 | 6 | 9 | 8 | 7 | 5 | 7 | 10 | 8 | 4 | 9 |
| Modularity (M) | 0.941 | 1.055 | 0.619 | 3.184 | 0.82 | 2.082 | 0.849 | 0.464 | 1.023 | 9.452 | 0.476 | 0.806 |

Table S3. The Spearman correlation between soil properties and soil microbial community in the alfalfa.

| Parameter | pH | EC | SOC | TP | AP | Actinobacteriota | Proteobacteria | Acidobacteriota |
| --- | --- | --- | --- | --- | --- | --- | --- | --- |
| pH | 1 | -.489** | -.787** | -0.171 | -.541** | 0.015 | 0.015 | .341* |
| EC |  | 1 | .714** | -0.024 | .241 | -0.16 | .235 | -0.157 |
| SOC |  |  | 1 | 0.111 | .514** | -0.023 | .177 | -.334** |
| TP |  |  |  | 1 | .225 | -.412** | -0.104 | 0.027 |
| AP |  |  |  |  | 1 | -0.099 | -0.026 | -0.108 |
| Actinobacteria |  |  |  |  |  | 1 | .636** | 0.008 |
| Proteobacteria |  |  |  |  |  |  | 1 | -.001 |
| Acidobacteriota |  |  |  |  |  |  |  | 1 |

**Continued**

| Parameter | Chloroflexi | Firmicutes | Myxococcota | Bacteroidota | Gemma-  timonadota | Methy-  lomirabilota | Plancto-  mycetota | Entoth-  eonellaeota |
| --- | --- | --- | --- | --- | --- | --- | --- | --- |
| pH | .383** | 0.209 | 0.136 | -.369** | .401** | 0.274** | 0.246 | 0.254 |
| EC | -.367* | -0.158 | -0.154 | .312* | -.203 | -0.225 | -0.127 | -0.047 |
| SOC | -.440** | -0.117 | -0.071 | 0.413** | -.290* | -0.251 | -0.281* | -0.083 |
| TP | 0.015 | .351** | .148 | -0.098 | .265* | 0.18 | -0.131 | .395** |
| AP | -0.237 | -0.149 | -0.064 | .297* | -.272* | -0.234 | -0.053 | -0.156 |
| Proteobacteria | 0.196 | 0.118 | .698** | .561** | .343** | 0.104 | 0.085 | .362** |
| Acidobacteriota | | .678** | | --- | | 0.068 | 0.102 | -0.174 | .313* | .292* | .739** | 0.239 |
| Chloroflexi | 1 | 0.144 | .322* | -.275* | .587** | .563** | .605** | .451** |
| Firmicutes |  | 1 | 0.543** | -0.13 | .621** | .543** | -0.157 | .644** |
| Myxococcota |  |  | 1 | 0.295** | .673** | .476** | -0.006 | .685** |
| Bacteroidota |  |  |  | 1 | -0.280* | -.418** | 0.065 | -0.127 |
| Gemmatimonadota |  |  |  |  | 1 | 0.743** | -0.033 | .841** |
| Methylomirabilota |  |  |  |  |  | 1 | 0.047 | .768*** |
| Planctomycetota |  |  |  |  |  |  | 1 | -.0.024 |
| Entotheonellaeota |  |  |  |  |  |  |  | 1 |

Note: soil properties (pH value, soil electrical conductivity, soil organic carbon, soil total phosphorus, and soil available phosphorus) among different alfalfa cultivars (*P ≤ 0.05, ** P ≤ 0.01).
